# Systematic Selection Signature Analysis of Chinese Gamecocks Based on Genomic and Transcriptomic Data

**DOI:** 10.3390/ijms24065868

**Published:** 2023-03-20

**Authors:** Xufang Ren, Zi Guan, Xiurong Zhao, Xinye Zhang, Junhui Wen, Huan Cheng, Yalan Zhang, Xue Cheng, Yuchen Liu, Zhonghua Ning, Lujiang Qu

**Affiliations:** National Engineering Laboratory for Animal Breeding, Department of Animal Genetics and Breeding, College of Animal Science and Technology, China Agricultural University, Beijing 100193, China

**Keywords:** Chinese gamecock, genome-wide association study, *F*_ST_, transcriptomic analysis

## Abstract

Selection pressures driven by natural causes or human interference are key factors causing genome variants and signatures of selection in specific regions of the genome. Gamecocks were bred for cockfighting, presenting pea-combs, larger body sizes, stronger limbs, and higher levels of aggression than other chickens. In this study, we aimed to explore the genomic differences between Chinese gamecocks and commercial, indigenous, foreign, and cultivated breeds by detecting the regions or sites under natural or artificial selection using genome-wide association studies (GWAS), genome-wide selective sweeps based on the genetic differentiation index (*F*_ST_), and transcriptome analyses. Ten genes were identified using GWAS and *F*_ST_: *gga-mir-6608-1*, *SOX5*, *DGKB*, *ISPD*, *IGF2BP1*, *AGMO*, *MEOX2*, *GIP*, *DLG5*, and *KCNMA1*. The ten candidate genes were mainly associated with muscle and skeletal development, glucose metabolism, and the pea-comb phenotype. Enrichment analysis results showed that the differentially expressed genes between the Luxi (LX) gamecock and Rhode Island Red (RIR) chicken were mainly related to muscle development and neuroactive-related pathways. This study will help to understand the genetic basis and evolution of Chinese gamecocks and support the further use of gamecocks as an excellent breeding material from a genetic perspective.

## 1. Introduction

Human- or nature-driven selection during domestication and breed formation over time can leave signatures in specific regions of the genome, such as increased allele frequencies, extensive linkage disequilibrium, homozygous genotypes, and decreased local diversity [1,2,3,4] Chinese gamecocks were artificially selected owing to the popularity of ancient Chinese cockfighting around 2500 BC [5]. This practice resulted in some unique morphological, physiological, and behavioural signatures that differentiate Chinese gamecocks from other chickens, such as different shaped combs, a larger body size, stronger limbs, and more aggression [6]. Besides being used for entertainment, foreign gamecocks have also been used as genetic material for the breeding of commercial breeds such as the Cornish, a famous modern broiler breed. Chinese gamecocks have similar characteristics to foreign gamecocks; however, few studies have reported on the genetic basis of Chinese gamecocks, such as systematic selection signature analysis during evolution and breeding. 

Identifying selection signatures associated with a phenotype will help reveal the processes that cause diversity among breeds. With the development of sequencing and genotyping technologies, different statistical tests have been conceptualised to detect selection signatures based on allele counts or allele frequencies. Genome-wide selective sweep analyses, including genome-wide association studies (GWAS) [7,8] and approaches based on the genetic differentiation index (*F*_ST_) [7,9], have been widely utilised to identify variants and genomic regions under selection. High-throughput transcriptomic analysis, which involves RNA sequencing (RNA-seq), has also been widely used to discover transcriptomic differences in a variety of tissues related to important traits of chickens [10,11]. It is generally believed that most complex traits are regulated by a network of genes, each associated with minor effects and environmental factors. A combination of genome-wide and RNA-seq data would help elucidate the pathways associated with complex traits and underlying genetic determinants [12,13,14].

There are currently five Chinese gamecock breeds according to the geographical location, the Henan, Luxi, Turpan, Xishuangbanna, and Zhangzhou. Signature selection based on Xishuangbanna gamecocks showed that 413 candidate genes were found to be putatively under selection, and these genes were related to traits, such as growth, disease resistance, aggressive behaviour and energy metabolism, as well as the morphogenesis and homeostasis of many tissues and orggans [15]. Reports on signature selection of Luxi and Turpan gamecocks suggested that some candidate genes associated with the nervous system, aggressive behaviour, and muscular development, including *AGMO*, *MEOX2*, *ISPD*, *BDNF*, could be detected [9]. More comprehensive research on Chinese gamecocks is needed.

In the present study, 603 chickens, including 54 Chinese gamecocks, were genotyped using the 600 K SNP Affymetrix Axiom Chicken Genotyping Array. Transcriptome data from muscle, hypothalamus, and testis samples from four adult male Luxi (LX) gamecocks and three adult male Rhode Island Red (RIR) chickens were analysed. Differentially expressed genes (DEGs) were identified and were used for gene ontology (GO) and Kyoto Encyclopaedia of Genes and Genomes (KEGG) analyses. The elucidation of the selection signature will enhance our understanding of the evolution and genetic basis of Chinese gamecocks and may support the further use of Chinese gamecocks as excellent native breeding material from a genetic perspective.

## 2. Results

### 2.1. Genome-Wide Putatively Selective Signatures in Gamecocks

**Figure 1:**
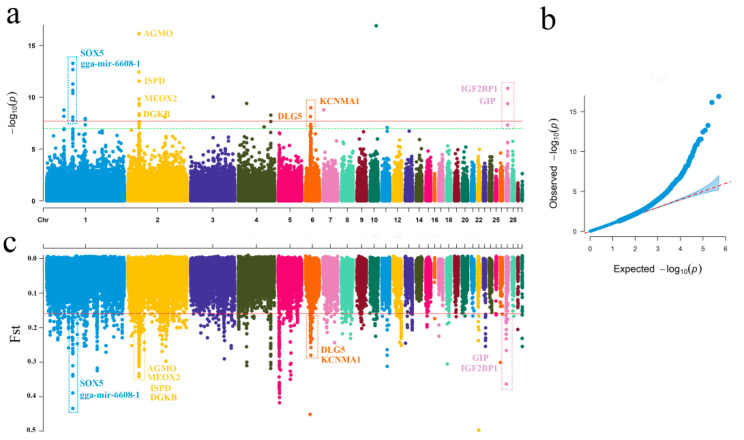
Selective signatures identified via GWAS and *F*_ST_. (**a**) Manhattan plot of GWAS values. The greenand red lines in the Manhattan plot represent genome-wide alpha level 1.00 × 10^−7^ and 2.00 × 10^−8^ adjusted based on the Bonferroni method with 498,363 SNPs after quality control, respectively. (**b**) Q-Q plot of GWAS values. Sudden separation of observed p values from expected p values indicate that traits are indeed affected by SNPs. (**c**) Distributionof Weir-*F*_ST_ values.

We performed GWAS analysis between five gamecock breeds and commercial, indigenous, foreign, and cultivated breeds. A total of 45 significant SNPs located on nine different chromosomes (1, 2, 3, 4, 6, 7, 10, 11, and 27) were identified, and 14 protein-coding candidate genes (*gga-mir-6608-1*, *SOX5*, *DGKB*, *AGMO*, *MEOX2*, *ISPD*, *IGF2BP1*, *GIP*, *SNF8*, *UBE2Z*, *EYA4*, *DLC1*, *DLG5*, and *KCNMA1*) were found in the regions 10 kb upstream and downstream of these SNPs (Table S1). The Manhattan and quantile-quantile (Q-Q) plots are shown in Figure 1a,b.

Using the top 1% Weir-*F*_ST_ value as the limit, signatures of selection were screened, and 126 genes were included (Table S2). Among these genes, ten genes (*gga-mir-6608-1*, *SOX5*, *DGKB*, *ISPD*, *IGF2BP1*, *AGMO*, *MEOX2*, *GIP*, *DLG5*, and *KCNMA1*) were selected using both approaches (Figure 1c). 

### 2.2. Overview of the RNA Sequencing Data

After filtering, the percentage of clean reads for each duplicate was over 96%. More than 90% of the bases were accurately identified with an error rate of 0.1%. The mapped ratios were greater than 87.5% for each library when the data were aligned with the reference genome (GRCg6a). These results confirmed that the RNA sequencing data qualified for subsequent analyses.

### 2.3. Identification of Differentially Expressed Genes

**Figure 2:**
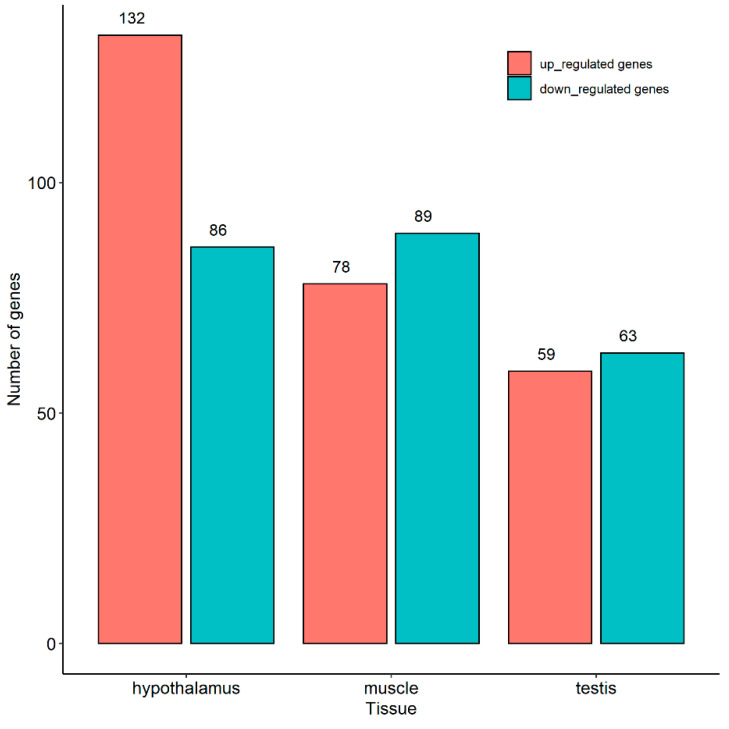
Differentially expressed genes in different tissues.

In total, 218, 167, and 122 DEGs were identified in the hypothalamus, muscle, and testis, respectively (Figure 2). Among these DEGs (Table S3), only *DGKB* and *KCNMA1* in the testis overlapped with candidate genes detected via GWAS and *F*_ST_ analyses. To some extent, this reflected the correction of the selection signature analysis.

### 2.4. Functional Annotation of Differentially Expressed Genes

**Figure 3:**
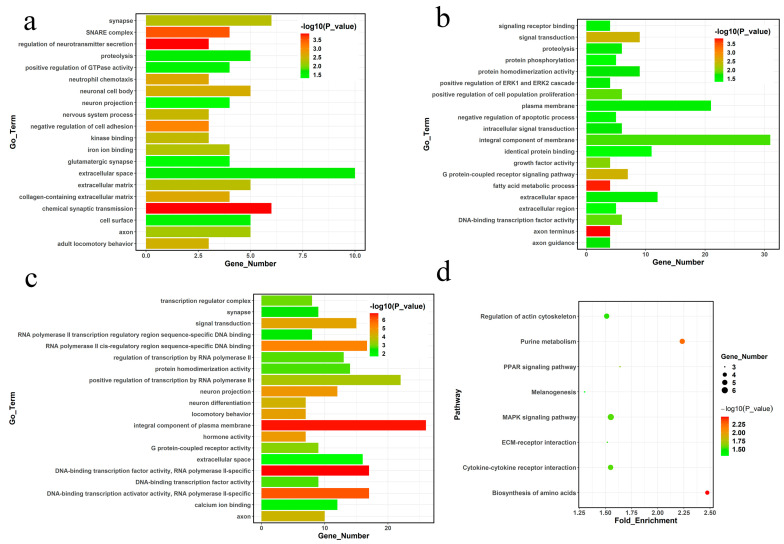
Functional analysis of DEGs. Significant GO terms of the (**a**) testis, (**b**) muscle, and (**c**) hypothalamus. (**d**) Top eight significant KEGG pathways of muscle.

GO enrichment analysis was performed to explore the functions of DEGs. The top 20 significant (*p <* 0.05) GO terms of the three tissues are shown in Figure 3a–c. In total, 166 significant GO terms were retrieved from the hypothalamus, including the integral component of the plasma membrane term (GO:0005887) and RNA polymerase II-related terms (GO:0045944, GO:0000978, GO:0000981, GO:0001228, and GO:0006357). Ninety GO terms were enriched in the testis, particularly the extracellular space term (GO:0005615), chemical synaptic transmission (GO:0007268), and some neuro-related terms (GO:0043025, GO:0030424, GO:0043005, and GO:0050877). Seventy-four significant terms related to cellular components (GO:0016021, GO:0005886, and GO:0005615) and signal transduction (GO:0007165) were retrieved from muscle. Detailed information is shown in Table S4.

Twelve significant pathways (*p <* 0.05) were enriched in the muscles. Two significant (*p <* 0.05) pathways (neuroactive ligand-receptor interaction and taurine and hypotaurine metabolism) were enriched in the hypothalamus, and only the steroid hormone biosynthesis pathway was significantly (*p <* 0.05) enriched in the testis (Table S5). The top eight significant pathways of the DEGs in muscle are shown in Figure 3d. These significant pathways revealed that Chinese gamecocks are different from other chicken types, especially regarding muscle development and neuroactive-related perspectives.

## 3. Discussion

The influence of genome selection can be detected by identifying variants and selection signatures. GWASs have mainly been used to detect genetic variants associated with various traits and diseases [16,17], and the *F*_ST_ statistic has been widely applied for the detection of signatures of selection [18,19]. In this study, we used a GWAS and *F*_ST_ to identify variants and selection signatures between Chinese gamecocks and commercial, indigenous, and foreign chicken breeds. Ten genes were identified using two approaches. Among the 10 genes, except *DLG5*, which has been proposed to play a role in cell signalling and cytoskeleton maintenance [20], the other nine genes were directly or indirectly related to some characteristics of the Chinese gamecock. 

A pea-comb is an adaptive trait that exists in almost all gamecocks. The small size of the comb reduces the chance of it being damaged or hurt during cockfighting. Copy number variation in intron 1 of *SOX5* is thought to be the cause of the pea-comb phenotype in chickens [21]. 

Gamecocks were bred for cockfighting and thus possess stronger muscles and skeletons to support the running and jumping necessary for fighting. *KCNMA1*, *MEOX2*, *ISPD,* and *MEOX2* are good candidates for muscle and skeletal development. *KCNMA1* encodes large-conductance voltage and Ca^2+^-activated K^+^ channels that are fundamental to the control of smooth muscle tone and neuronal excitability [22,23,24]. *MEOX2* and *ISPD* also play crucial roles in muscle and skeletal development [25,26,27]. *IGF2BP1* is a well-known gene that plays an important role in the body size and weight of chickens and ducks [28,29]. In addition, the major regulators of skeletal muscle development [30] the mitogen-activated protein kinase (MAPK) signalling pathway, were enriched in the gamecock muscle.

Compared to other types of chickens, gamecocks suffer more damage due to their use in cockfighting. We, therefore, speculated that some immune-related genes remained in the gamecocks over time to help them recover quickly. The detection of *AGMO*, which has been reported to be associated with inflammation, proved our speculation in some way [31]. 

Large amounts of energy are essential for cockfighting, and glucose provides instantaneous and efficient energy for cellular metabolism. To this point, two genes identified in our study are thought to be associated with glucose metabolism, *DGKB* [32] and *GIP*, which maintain glucose homeostasis by encoding proteins [33]. 

In the hypothalamus, several terms were associated with RNA polymerase II-related functions. This suggests that additional mRNA was synthesised to provide sufficient protein for gamecocks, including different hormones, such as thyrotropin-releasing hormone and gonadotropin-releasing hormone. KEGG annotation suggested that neuroactive ligand-receptor interactions, as well as taurine and hypotaurine metabolism pathways, were enriched. The neuroactive ligand receptor interaction pathway includes all ligand receptors on the plasma membrane that are associated with intracellular and extracellular pathways, and previous research has shown its important role in stress responses [34,35,36,37]. The taurine and hypotaurine metabolic pathways are considered the major pathways of cysteine metabolism in astroglial cells [38]. Taurine is involved in the regulation of intracellular calcium concentrations [39] and plays cytoprotective and developmental roles, especially in neurological and ocular tissues [40]. Thus, we propose that the taurine and hypotaurine metabolism pathways contribute to the vision and sharper reflexes of the gamecock during cockfighting.

GO terms in the testis were mainly enriched for neuroactive-related functions. The KEGG analysis suggested that only the steroid hormone biosynthesis pathway was enriched. Testosterone is an important androgen that is produced via the steroid hormone biosynthesis pathway. The role of testosterone is mainly manifested in cognitive functions [41], muscle mass and power [42], and mood adjustments [43]. Therefore, these aspects may be closely related to the aggressive behaviour of gamecocks.

## 4. Materials and Methods

### 4.1. Sample Collection and SNP Genotyping

A total of 603 adult chickens, including gamecocks, were randomly selected from 20 chicken breeds. Detailed information on these samples is presented in Table 1.

A total of 86 blood samples were collected from the Big Bone, Chahua, Turpan Game, Zhangzhou Game, Xishuangbanna Game, Henan Game, Luxi Game, and Wenchang breeds and sent to Neogene Biotechnology Co., Ltd. (Shanghai, China) for 600 K microarray genotyping sequencing. Microarray data for the other 517 samples were collected in our laboratory. Genotyping was performed using the 600 K Affymetrix Axiom Chicken Genotyping Array (Affymetrix, Inc., Santa Clara, CA, USA), which included 580,961 SNPs across the entire chicken genome. After genotyping, a ped-and-map file was obtained. SNP filtering for each individual was performed using PLINK (version 1.90) [44]. After removing SNPs from the sex chromosome, autosomal SNPs were filtered if an individual’s SNP deletion rate was greater than 0.1 (using the “--mind” command), the SNP deletion rate of a single locus was greater than 0.1 (using the “--geno” command), and the minor allele frequency of SNP loci was less than 0.05 (using the “--maf” command). After quality control, information on 498,363 high-quality SNPs and 593 individuals, including 52 gamecocks, remained in binary files (bed, bim, and fam) for GWAS and *F*_ST_ analyses.

### 4.2. Genome-Wide Association Studies

In this study, on one hand, the comb shape of five gamecock breeds is pea-comb, the comb shape of Dongtao chicken and Taihe Silky chicken is strawberry comb, and the comb shape of the remaining thirteen breeds is single comb. On the other hand, gamecocks were domesticated for the special use of cockfighting activity and finally formed the independent breeds; thus, the aggression of gamecocks is generally agreed to be stronger than that of other chickens. Considering the differences between Chinese gamecocks and other chicken breeds are mainly reflected by qualitative characteristics, such as shape of the comb and aggression, we performed a case-control GWAS analysis. Five gamecock breeds were used as the experimental group, and all other individuals were used as the control group. Association analyses were performed using GEMMA software (version 0.98.1) [45,46] based on the linear mixed model (LMM) association algorithm. Considering that the validity of a GWAS can be affected by population stratification [47], a principal component analysis (PCA) was performed, and different numbers of principal components (PCs) were added as covariates to correct the results of the association analysis; however, the original inflation factor (λ = 0.952) was the best value compared with the added PCs. This might be because the linear mixed model effectively controlled population stratification. Thus, the original result that did not include PCs as covariates remained. The Manhattan and Q-Q plots for GWAS were generated using the CMplot package (https://github.com/YinLiLin/CMplot, accessed on 4 October 2022) in R. All *p*-values were adjusted for multiple testing using the Bonferroni method with alpha levels of 1.00 × 10^−7^ and 2.00 × 10^−8^. Combined with the chicken reference genome (Gallus_gallus-5.0) released by Ensemble, upstream and downstream 10 kb sequences of the SNPs with *p*-values less than 1.00 × 10^−7^ were aligned to the corresponding genes using the Ensemble Biomart online tool (http://oct2018.archive.ensembl.org/biomart/martview/2170f2f8151a7ba30481e705052baee0, accessed on 19 September 2022).

### 4.3. Genome-Wide Selective Sweep Analysis

Binary files were converted to vcf format using PLINK software (version 1.90). *F*_ST_ was then used to identify the genomic regions under selection in the gamecocks. The window size and step size were set to 20 kb and 10 kb, respectively, to calculate the Weir-*F*_ST_ value using the vcftools software (version 0.1.13). Weir-*F*_ST_ values in the top 1% were regarded as putative genomic regions that were under selection. Annotation of the regions of interest was implemented using the Ensemble Biomart online tool (http://oct2018.archive.ensembl.org/biomart/martview/2170f2f8151a7ba30481e705052baee0, accessed on 19 September 2022). The results were visualised using the CMplot package in R.

### 4.4. RNA Sequencing

Considering that aggression in chickens is mainly reflected in male individuals, we collected muscle, hypothalamus, and testis samples from four adult male LX gamecocks and three adult male RIR chickens. Tissue samples were placed in RNA preservation solution, stored at −20 °C and sent to ANOROAD (Beijing, China) for further use. Total RNA was extracted from tissue samples using TRIzol reagent. The RNA purity was measured using a NanoDrop spectrophotometer. Concentration and integrity were measured using an Agilent 2100 Bioanalyzer and Agilent RNA 6000 Nano Kit, respectively. Pair-end sequencing (150 bp) was performed using an Illumina Novaseq 6000 platform by a commercial service company. High-quality reads were obtained from the company after removing low-quality base ratios (Q ≤ 19) greater than 50%, raw reads with adapter pollution, and N ratios greater than 5%, with Q30 as the evaluating indicator.

### 4.5. Analysis of Differentially Expressed Genes

Clean reads were aligned against the chicken reference genome (GRCg6a) using HISAT2 software (version 2.2.0). FeaturesCounts software (version 1.5.2) was used to construct the expression matrix. After the results of FeaturesCounts were returned, differential expression analysis was performed using DESeq2 (Version 1.36.0) in R [48]. The criteria for identifying DEGs were |log2FoldChange| > 1 and padj < 0.05. The DEGs were annotated using the Ensemble Biomart online tool (http://oct2018.archive.ensembl.org/biomart/martview/2170f2f8151a7ba30481e705052baee0, accessed on 20 September 2022). With the name of DEGs as input and *p* < 0.05 as the significance level, GO analysis of the differentially expressed target genes and KEGG pathway analysis for these genes were conducted using KOBAS (http://kobas.cbi.pku.edu.cn/kobas3, accessed on 23 September 2022) and visualised in R. 

## 5. Conclusions

We identified 10 genes (*gga-mir-6608-1*, *SOX5*, *DGKB*, *ISPD*, *IGF2BP1*, *AGMO*, *MEOX2*, *GIP*, *DLG5*, and *KCNMA1*) in the Chinese gamecock using a combination of GWAS and the *F*_ST_-based approaches. These genes are mainly associated with muscle and skeletal development, glucose metabolism, and the pea-comb phenotype. The enrichment results of DEGs showed that differences between the LX gamecock and RIR chickens were reflected by muscle development and neuroactive-related pathways. This study has been helpful in understanding the evolution and genetic basis of Chinese gamecocks for chicken breeders at home and abroad and may support the further use of gamecocks as excellent native breeding material from a genetic perspective. However, further study on the roles of these candidate genes during the evolution and breeding of Chinese gamecocks is still required.

## Figures and Tables

**Table 1:** Detailed information of the chicken samples used in our study.

Population/Breed	Geographic Origin	Classification	Number
Henan Game	Henan Province, China	Fight breed	13 F
Luxi Game	Shandong Province, China	Fight breed	2 M/8 F
Turpan Game	Xinjiang Province, China	Fight breed	6 M/5 F
Xishuangbanna Game	Yunnan Province, China	Fight breed	10
Zhangzhou Game	Fujian Province, China	Fight breed	10
Big Bone	Liaoning Province, China	Indigenous breed	5 M/5 F
Beijing You	Beijing, China	Indigenous breed	50
Chahua	Yunnan Province, China	Indigenous breed	5 M/6 F
Hongshan	Hubei Province, China	Indigenous breed	24 M/24 F
Piaoji	Yunnan Province, China	Indigenous breed	9F
Shouguang	Shandong Province, China	Indigenous breed	50
Taihe Silky	Jiangxi Province, China	Indigenous breed	50
Tibetan	Tibet, China	Indigenous breed	40
Wenchang	Hainan Province China	Indigenous breed	11
RIR	Rhode Island, America	Commercial breed	50
WhiteLeghorn	Tuscany, Italy	Commercial breed	40
Dwarf Layer	Guizhou Province China	Cultivated breed	50
Dwarf Yellow Broiler	Guizhou Province China	Cultivated breed	50
Dongtao	Vietnam	Foreign breed	20 M/10 F
Houdan	France	Foreign breed	50
Total			603

Note: F means female. M means male. Individuals with neither F nor M have missing sex information.

## Data Availability

The datasets used in this study are available from the corresponding author upon reasonable request.

## Supplementary Materials

The supporting information can be downloaded at: https://www.mdpi.com/article/10.3390/ijms24065868/s1.
